# COVID-19, economic threat and identity status: Stability and change in prejudice against Chinese people within the Canadian population

**DOI:** 10.3389/fpsyg.2022.901352

**Published:** 2022-10-28

**Authors:** Victoria Maria Ferrante, Éric Lacourse, Anna Dorfman, Mathieu Pelletier-Dumas, Jean-Marc Lina, Dietlind Stolle, Roxane de la Sablonnière

**Affiliations:** ^1^Department of Psychology, Université du Québec à Montréal, Montreal, QC, Canada; ^2^Department of Sociology, Université de Montréal, Montreal, QC, Canada; ^3^Department of Psychology, Bar-Ilan University, Ramat Gan, Israel; ^4^Department of Psychology, Université de Montréal, Montreal, QC, Canada; ^5^Department of Electrical Engineering, École de Technologie Supérieure de Montréal, Montreal, QC, Canada; ^6^Department of Political Science, McGill University, Montreal, QC, Canada

**Keywords:** prejudice, economic threat, identity, pandemic, longitudinal

## Abstract

**Objectives:**

Previous studies found a general increase in prejudice against Chinese people during the first months of the pandemic. The present study aims to consider inter-individual heterogeneity in stability and change regarding prejudice involving Chinese people during the pandemic. The first objective is to identify and describe different trajectories of prejudice over a seven-month period during the pandemic. The second and third objectives are to test the association between trajectory group membership and antecedent variables such as: socio-demographic factors (i.e., age, gender, political affiliation) and two psychological mechanisms, namely economic threat and global citizenship identification.

**Methods:**

A representative Canadian sample (*N* = 3,617) according to age, gender and province of residence, was recruited for a 10-wave survey starting from April 2020 to December 2020. First, a group-based modeling approach was used to identify trajectories of prejudice. Second, a multinomial logistic regression model was used to test associations between membership in trajectories and antecedents.

**Results:**

Four trajectories were identified. The first three trajectories have a low (71.4% of the sample), high (18.5%) or very high (5.3%) level of prejudice *against* Chinese people which is relatively stable over time. The fourth trajectory (4.9%) reports low levels of prejudice *in favor* of Chinese people which become more positive throughout 2020. Regarding socio-demographic factors: gender is not associated with trajectory group membership, younger people are more likely to follow the trajectory in favor of Chinese people and conservatives are more likely to follow the highest trajectories against Chinese people. Regarding some psychological mechanisms: personal but not collective economic threat is associated with the trajectory in favor of Chinese people. Finally, the highest levels of prejudice are found when the strategy of identification is more local rather than global.

**Conclusion:**

The present study shows that Canadians differ in terms of both their level and change in prejudice against Chinese people throughout the pandemic with some socio-demographic groups being more likely than others to be associated with prejudice. The results also suggest that a promising way to tackle the major social issue of prejudice is to highlight a vision of the world where individuals are all “global citizens” facing the same challenge.

## Introduction

The COVID-19 pandemic first identified in Wuhan, China, has recently transformed societies and the lives of billions of individuals. Since Chinese people are often held responsible for the pandemic (Borja et al., 2020; Jackson et al., 2020), they could have been affected by a parallel “racial epidemic” (Pang, 2021, p. 235). As an illustration, many media outlets and authors evoked the emergence of an international and global “anti-Chinese sentiment” (Kang, 2020; Vachuska, 2020; White, 2020).

This negative sentiment towards individuals or groups, strictly based on the group membership, refers to “prejudice” (Leyens and Yzerbyt, 1997; Leyens, 2020). Many previous studies on prejudice against Chinese people during the pandemic are cross-sectional and do not investigate stability or change in prejudice over time. Some studies considered multiple measurement time points to directly address if there was indeed an increase in prejudice. These prospective studies showed that anti-Chinese slurs increased on the internet during the first months of the pandemic (Nguyen et al., 2020; Schild et al., 2020; also see Vachuska, 2020).

As the pandemic entails specific factors that can impact prejudice, there is a need for intensive longitudinal studies to investigate if prejudice will increase, crystallize or decrease as the crisis continues. Most importantly, additional studies need to verify the external validity of past findings as they tended to assume that the noted increase in prejudice was embraced by a homogeneous population. Indeed, it has long been theorized that, while facing the same context, inter-individual differences occur during the formation and maintenance of prejudice, leading to different baseline levels and patterns of change across individuals with some individuals remaining stable while others decreasing their levels of prejudice (Foley, 1974). These inter-individual differences in levels and evolution of prejudice should be taken into account when it comes to understanding the dynamics of prejudice, especially in such an unprecedented, lasting crisis like the COVID-19 pandemic.

The main goal of this paper is to determine whether in a given population, individuals would react in the same, homogeneous way regarding the level of change in their prejudice against Chinese people during the pandemic or would several patterns of prejudice be observed. Can the population be divided into homogeneous subgroups? With this perspective in mind, it is important for longitudinal research to be conducted among large, representative samples of their studied populations in order to ensure that the proportion of each eventual subgroup could be adequately estimated. The present study benefits from such empirical data, because it followed more than 3,500 Canadians adults with similar age, gender, and province of residence to those of the Canadian adult population over the first months of the pandemic.

The first objective aims to identify and describe the different trajectory groups of prejudice. This new contribution will allow us to reach a deeper, more acute understanding of the dynamics of prejudice against Chinese people during the pandemic by answering the following questions: Did the level of prejudice change over time for some groups of individuals? If yes, for whom did it change?

Distinguishing if there are one or more subgroups of prejudice among a given population is the first step to fully understand the dynamics of prejudice during the COVID-19 pandemic. Next, understanding what can fuel or lessen the different patterns of prejudice in this specific context may have important practical and theoretical implications. Our second objective aims to test the association between the trajectory group membership of prejudice and “traditional” socio-demographic factors that have already been associated with prejudice against Asian people during the pandemic (i.e., age, gender, political affiliation). Finally, our third objective aims to test the association between the trajectory group membership of prejudice and two psychological mechanisms prominent in the socio-psychological literature and known to be relevant within a context of social change: economic threats (i.e., relative deprivation theory) and identity status (i.e., global versus local citizenship identification).

### COVID-19 and the increase in prejudice against Chinese people

When COVID-19 was recognized as a pandemic by the World Health Organization on March 11, 2020, there were already more than 121,000 infections (Faucher et al., 2022) and 4,500 deaths worldwide (Ritchie et al., 2020). Global daily infections increased rapidly and drastically throughout 2020 while the number of deaths reached 1.88 million at the end of December 2020, with almost 16,700 occurring in Canada (Ritchie et al., 2020). Unlike China or the United States, Canada was less impacted by the pandemic because the country reacted quickly to stop the spread of the virus with, for example, the closing of the borders on March 17, 2020, the successful mobilization of the population regarding adherence to health measures, and several other factors that allowed the health system not to become overwhelmed (Detsky and Bogoch, 2020; Ferrante et al., 2020a, b). The rate of increase in daily infections was low compared to other countries, with even a decline in cases from May 4 to July 13, 2020 (i.e., waves 3 to 7 in the present study) before increasing again but weakly until late December 2020 (Ritchie et al., 2020).

In the presence of a pathogen, individuals tend to develop prejudice against certain groups without even being aware of it, as they tend for example to avoid people in contact with the virus to protect themselves (i.e., behavioral immune system theory, Schaller, 2011; Schaller and Park, 2011; Taylor, 2019). Since the belief in a pathogen-ethnicity link is strongly rooted in the collective imagination (Gee et al., 2020), foreigners have often been targets of prejudice during previous epidemics, especially Chinese people (White, 2020; Jones, 2021). The COVID-19 pandemic was no exception and its association with China has even been this time directly established and mediatized (i.e., the so-called “Chinese Virus”).

Consequently, a lot of behavioral incidents directed against Chinese people and generally against Asians have been reported globally during the COVID-19 pandemic. International surveys and studies indeed corroborate the fact that Asian people were particularly discriminated against due to the pandemic (Cheah et al., 2020; Ipsos MORI, 2020; Ruiz et al., 2020; Lee and Waters, 2021). As a result, the increasingly “anti-Chinese sentiment” assuming to underly such behavioral incidents became an axiom instead of a research avenue. Thus, there is a lack of research on prejudice against Chinese people within the broad COVID-19 pandemic research.

Even among the rare studies that did focus on prejudice, “anti-Chinese sentiment” is assumed to have increased within the general population. Thus, many of these studies focused on predictors of the prevalence or frequency of prejudice (see Croucher et al., 2020; Dhanani and Franz, 2020a, b; Tsai et al., 2020). For example, Dhanani and Franz (2020a, b) showed using a cross-sectional design in the United States, that factors such as fear of COVID-19, lack of knowledge about the virus, lack of trust in science and trust in the American ex-president (i.e., Donald Trump) were positively associated with prejudice against Chinese and Asian people. In another experimental study conducted among American adults, the same authors manipulated three factors in a statement related to the pandemic: (1) the fact that the coronavirus emerged in China or consisted in a mutation unrelated to China, (2) the severity of economic threats due to the pandemic, and (3) the severity of the health threat engendered by the coronavirus. They found that focusing on the Chinese origin of the virus and on the economic threat of the virus caused prejudice against Asian people while the health threat did not (Dhanani and Franz, 2021).

Although the investigation of predictors or causal factors related to the pandemic is necessary in order to understand what may fuel or lessen prejudice in individuals in this specific context, neither cross-sectional nor experimental studies inform us about change in prejudice over the course of the pandemic. Yet, one cannot just assume that prejudice increased during the pandemic based on media reporting or research on related-phenomena (e.g., discrimination). The only way to know if anti-Chinese manifestations are also increasingly happening on a cognitive and affective level is to investigate change in self-reported prejudice. In summary, it is essential to consider the possibility for prejudice to change (e.g., increase, decrease) throughout the pandemic.

The other previous studies are more informative regarding change in prejudice following the pandemic. By using a long-term perspective with data extracted from the internet, they addressed the question: Did prejudice increase over time? For example, the American Google Trends’ analysis of Vachuska (2020) conducted from February 2020 to June 2020 shows that searches on pandemic-related information correlated both with an increase in anti-Chinese slurs and a disinterest toward Chinese restaurants. In the same vein, another international study conducted from November 2019 to March 2020 demonstrated that the use of anti-Chinese slurs was more widespread and diversified over time on social media such as Twitter (Budhwani and Sun, 2020; Nguyen et al., 2020; also see Schild et al., 2020). In summary, these studies aimed to understand whether prejudice against Chinese people increased during the pandemic or not, indeed confirming such an increase during the first months of the pandemic.

The major contribution of these longer-term prospective studies is their interest regarding stability and change in prejudice during the pandemic. By showing that prejudice increased following the first months of the pandemic, they support the general assumption of a growing “anti-Chinese sentiment,” highlighting a social issue that needs to be addressed. Nevertheless, the interpretation of their results should be nuanced, as the increase in observed prejudice cannot necessarily be generalized to the entire population. First, these studies may have an important selection bias given the fact that their data was indirectly extracted from the internet. Second, the approach used by these studies reveals that prejudice increased on average, as a general tendency within a given population. It does not mean that everyone in the population embraced this increase in prejudice. There were for example many supportive individuals and organizations that helped the Chinese community during the pandemic (Macguire, 2020; Chan, 2021; Lui et al., 2021). Thus, asking whether there was a general increase in prejudice or not over the course of the pandemic is not sufficient. The next step is to better understand who exactly is concerned with this increase in prejudice and to determine if other systematic patterns of change in prejudice could be identified.

The present study proposes an in-depth investigation of the dynamics of prejudice against Chinese people during the pandemic within the Canadian adult population. Such an investigation requires taking into account inter-individual heterogeneity within a given population, as individuals could report a different pattern of change in prejudice over time. These patterns of change in prejudice can also be referred to as a “trajectory” of change.

### COVID-19 and the heterogeneity in trajectories of prejudice

The first objective of the present study proposes to identify the heterogenous trajectories of prejudice against Chinese people during the COVID-19 pandemic. This new theoretical and methodological perspective examined by the present study in the literature of prejudice is based on the assumption that when it comes to understanding the dynamics of prejudice within a given population, inter-individual differences should be taken into consideration. In other words, a population is not a homogeneous entity regarding both prejudice levels and change. Inter-individual differences are thus expected to be revealed at two levels: a difference in the initial level of prejudice may be observed as well as a difference in the degree of stability/change (i.e., linear, quadratic or more complex polynomials).

#### Heterogeneity in initial levels of prejudice

Regarding the initial level of the intensity of prejudice, the literature indeed assumes that some individuals have low levels of prejudice while others have high levels against any groups (Lindzey, 1950; Allport, 1979). In addition, these inter-individual differences led to the first theories which explain heterogeneity in levels of prejudice by personality traits (i.e., the authoritarian personality theory, Adorno et al., 1950; the scapegoat theory of intergroup conflict, Leyens and Yzerbyt, 1997). Such a perspective implied a certain within-person stability as some individuals would be predisposed to remain consistently prejudiced while others would be predisposed to remain less or non-prejudiced. That is to say, individuals would distinguish themselves based on their basic, initial level of prejudice, regardless of their change in prejudice over time.

Taking into consideration this theoretical notion of systematic inter-individual differences regarding basic levels of prejudice has important implications when it comes to the study of change in prejudice. Indeed, individuals who report different levels of prejudice during a first observation may also report different patterns of change in prejudice over the course of the following observations. For example, Foley (1974) found that while facing the same context of interracial contact, the initially lowest prejudiced individuals did not change in their prejudice while the initially highest prejudiced individuals were those who reported the biggest decrease in prejudice. The author concludes about the possibility for prejudice to change over time, while specifying that the direction of change is a function of personality and environmental factors.

#### Heterogeneity in the evolution of prejudice

Regarding the evolution of prejudice over time, it requires recognizing that prejudice can change in different and even multiple directions (e.g., increase, decrease, curvilinear trajectory), but can also remain stable. Contrary to Foley’s findings, some authors argue, for example, that the more extreme the attitudes are, the more resistant they are to change (Eagly and Chaiken, 1995). There is support in the literature for both the stability and the malleability of prejudice over time (Akrami et al., 2009). While pioneering theories of prejudice focused on personality by explaining the likelihood to be prejudiced in a stable way, and considering prejudice as “a trait of personality” (Allport, 1979, p. 73), subsequent theories of prejudice explored the malleability of prejudice by investigating which situational factors lead to change in prejudice. For example, although the intergroup contact hypothesis assumes that intergroup contact reduces prejudice (for a meta-analysis, see Pettigrew and Tropp, 2006), it requires to take into consideration several conditions and factors for the reduction to occur (Allport, 1979; Pettigrew and Tropp, 2008).

While previous studies on the COVID-19 pandemic answered the question “Is there a change in prejudice towards Chinese people?” The current work considers both the stability and the malleability of prejudice raising the question: does everyone change, and if so, do they change in the same direction? This new contribution will further help to understand which individuals are the most vulnerable when it comes to developing and/or renforcing prejudice in the context of dramatic social changes, which are “the new normal” (de la Sablonnière, 2017, p. 2).

### COVID-19 and antecedents of prejudice heterogeneity

#### Socio-demographic factors

##### Age and gender

Some studies have been conducted to determine if low versus high prejudiced individuals differ regarding their socio-demographic characteristics (Allport and Kramer, 1946; Lindzey, 1950). Overall, studies contradict each other and did not lead to established conclusions, especially regarding “sex, age, [and] region” (Allport, 1979, p. 80). Such contradictory results were also reported in the COVID-19 pandemic context: some cross-sectional studies found that age and gender predict prejudice against Asian people with young people and women being less prejudiced than older people and men (Dhanani and Franz, 2020a, b, 2021), whereas Tsai et al. (2020) found that older people were less prejudiced against Asian people than younger people and that there was no difference between men and women.

However, none of these authors discussed gender differences, which probably reflects the literature’s lack of explanations about underlying mechanisms. Although studies consistently found women to be less prejudiced than men, this difference is assumed to stem from cultural rather than natural factors, such as institutional ones, as this difference is not observed among children (Allport and Kramer, 1946). Regarding age, studies suggest that prejudice increases with age (Henry and Sears, 2009), while rejecting the explanation of a “cohort effect.” Thereby, some authors argue for a cognitive explanation as older people would be less able of repressing their implicit (i.e., unconscious) prejudice while another sociocultural explanation posits that the increased levels of prejudice observed among older people could be explained by a greater endorsement of the right-wing authoritarian ideology (Franssen et al., 2013).

##### Political affiliation

The association between political affiliation and prejudice is well documented. Research consistently found conservatives to be more prejudiced than liberals (Lindzey, 1950; Allport, 1979) and these results have been replicated during the COVID-19 regarding prejudice against Chinese and Asian people (Tsai et al., 2020; Dhanani and Franz, 2020a, b, 2021).

Two theories have proposed some explanations about the tendency for conservatives to be more prejudiced than liberals (Kite and Whitley, 2016). According to the social dominance orientation (SDO) theory which found strong correlations between SDO construct and conservative political ideology, conservatives would endorse at a greater extent a hierarchical view of the social system, based on the ingroup’s power and dominance over lower-status groups (by opposition to an egalitarian view based on equality) that they would use as “legitimizing myths” to justify their prejudice and privileges. Regarding the attribution-value model (Crandall et al., 2001), prejudice results from the perception that members of minority groups present negative characteristics for which they are at fault. Since the notion of personal responsibility is more important for conservatives than liberals, they would attribute negative outcomes (e.g., their poverty) to minority groups rather than to other circumstances. As an illustration related to the COVID-19 context, Hardy et al. (2021) found that conservatives were more likely than liberals to blame China and Chinese people for being responsible for the pandemic.

Finally, research also used to support that conservatives are more likely sensitive to threat than liberals, as they would perceive the world as more dangerous and unpredictable (Van Leeuwen and Park, 2009). However, it has been nuanced, as Nail et al. (2009) showed that when liberals are exposed to some threats, they tend to become more conservative. But one could still wonder if the results of Nail et al. (2009)‘s studies might not depend on the type of threat assessed. Indeed, contemporary research on the link between political affiliation and threat suggests that some threats such as climate change would constitute a lesser concern for conservatives than liberals. In the COVID-19 pandemic context, Calvillo et al. (2020) observed, for example, that conservatives perceived the virus as less dangerous but were at the same time more likely to endorse conspiracy theories about the spread of the virus, which is an attitude linked to prejudice (Jolley et al., 2020; Douglas, 2021).

#### Psychological mechanisms

##### Economic threat

Economic insecurities or dissatisfaction constitute a major human concern which is often associated with an increase in prejudice (Allport, 1979; Stephan and Stephan, 2000, 2017). The relative deprivation theory (Crosby, 1976) offers a conceptualization of such an economic threat. In the present study, we will focus on economic temporal relative deprivation, both personal and collective. Economic temporal relative deprivation refers to the perception of a disadvantage between the current state of economic resources possessed by an individual (personal, referred in our study as personal relative deprivation) or her/his group such as Canadians (collective, referred as collective relative deprivation) compared to the individual’s own or group past situation. Added to this perception is an unpleasant feeling that results from this comparison over time as individuals feel themselves dispossessed by something they may otherwise deserve (Runciman, 1966; Albert, 1977; Walker and Pettigrew, 1984). It has been demonstrated that high levels of economic temporal relative deprivation are positively related to prejudice toward an outgroup (Dambrun and Guimond, 2001; Dambrun et al., 2006; de la Sablonnière et al., 2013).

The relative deprivation theory distinguishes itself from previous theories which consider an economic threat (e.g., integrated threat theory; Stephan and Stephan, 2000, 2017) by highlighting the idea that threat comes from the gap between two states rather than from the object of the threat itself. We assumed that relative deprivation theory is relevant regarding the pandemic context as this event happened with a “before” and an “after.” This theory is widely supported by research in predicting prejudice (Smith et al., 2012).

##### Identity status

Social identification refers to the process by which individuals feel themselves belonging to several significant ingroups by sharing characteristics with the other ingroup members (i.e., social identity theory, Tajfel, 1982). Social identification may occur at several levels, such as at a local “subgroup” level more or less inclusive (e.g., national identification) and at a global “supra ordinal” level of mankind, which is the most inclusive one (i.e., global citizenship identification) (i.e., self-categorization theory, Tajfel and Turner, 1986; Turner et al., 1987). Social identification is highly context dependent; in this perspective, if individuals can identify themselves with multiple groups to which they belong, recent studies suggests that social identification occurs one ingroup at a time as the multiple identities possessed by individuals remain cognitively separated (Caron-Diotte and de la Sablonnière, 2022). Specifically, literature suggests that local and global citizenship identification are two distinct and separated strategies of identification which serve different purposes and can lead to different outcomes notably in terms of prejudice (Gorman and Seguin, 2018; Jetten et al., 2020). At the very least, we might say that certain identities take precedence over others due to circumstances or situations.

In line with this theoretical perspective, Jetten et al. (2020) recognized several scenarios regarding identity status and prejudice within the pandemic context. On the one hand, individuals may identify themselves with a local ingroup in opposition to an outgroup likely to propagate the coronavirus such as the Chinese people which will result in an “us” versus “them” dynamic. Local identification is indeed a common strategy while facing an existential threat such as the coronavirus which systematically leads to denigration and prejudice against outgroups (i.e., terror management theory, Greenberg et al., 1986; Greenberg and Kosloff, 2008). Literature suggests that higher levels of local identification are positively associated with prejudice (Tajfel and Turner, 1986; Castano et al., 2002; Bourhis, 2020). On the other hand, individuals may identify themselves globally, as citizens of the world. This type of identification referred in the present study as “global citizenship identification” can be situationally activated or stem from individual differences, with some individuals having a greater tendency than others to identify themselves as global citizens (Hamer et al., 2019). According to the common identity model (Dovidio et al., 2000) which uses the situational approach (Hamer et al., 2019), the more inclusive the ingroup identification, the less prejudice there is (Gaunt, 2009; Leyens, 2015). Since global citizenship identification is the ultimate level of inclusiveness, literature supports that it is associated with less intergroup bias such as prejudice (McFarland, 2017; Sparkman and Eidelman, 2018) with a greatest valuation on human rights and lives (McFarland et al., 2019; Sparkman and Hamer, 2020). The beneficial outcomes of global citizenship identification have even been studied in this unprecedented, global context of the COVID-19 pandemic, suggesting that compared to local identification, global citizenship identification was associated with pro-social behaviors aiming at preventing the propagation of the virus (Barragan et al., 2021; Marchlewska et al., 2022; Sparkman, 2022). In this paper, we refer to “local identification” at a national level (i.e., Canadian citizens).

## Overview of the present study and hypotheses

The present study is part of the project: “COVID-19 Canada: The end of the world as we know it?” (“COVID-19 Canada: La fin du monde tel qu’on le connaît?,” in French), which was approved by the Ethic Committee for Research in Education and Psychology at the Université de Montréal. This project consists of a large national and longitudinal 12-waves survey spanning from April 2020 to April 2022. Readers interested in obtaining detailed information about the survey and its methodology can consult the technical reports (de la Sablonnière et al., 2020a; Caron-Diotte et al., 2021).

The present study focuses on the first year of the pandemic with data from waves 1 to 10 (early April 2020 to late December 2020). Our three objectives are (1) to identify trajectory groups of prejudice against Chinese people, (2) to test the association between the membership of these trajectory groups and socio-demographic factors, and (3) to test the association between the membership of these trajectory groups and two COVID-19-related psychological mechanisms.

*Hypothesis 1*: There are different trajectory groups of prejudice against Chinese people during the COVID-19 pandemic which differ regarding their initial level of prejudice (e.g., low, medium, high) and their change over time (e.g., increase, stagnation, decrease).

*Hypothesis 2*: Trajectory group membership of prejudice is associated with socio-demographic factors. Regarding political affiliation, it is expected that conservatives will be more likely to belong to high trajectory groups of prejudice than liberals (H2a). Regarding age and gender, we do not make any specific sub-hypothesis.

*Hypothesis 3*: Trajectory group membership of prejudice is associated with economic threat. More specifically, it is expected that participants who report more (H3a) personal relative deprivation and (H3b) collective relative deprivation will be more likely to belong to high trajectory groups of prejudice.

*Hypothesis 4*: Trajectory group membership of prejudice is associated with identification status. More specifically, it is expected that participants who report a more local identification over a global one will be more likely to belong to high trajectory groups of prejudice.

## Materials and methods

### Participants and procedure

Tables 1, 2 provide information about the sample’s socio-demographic characteristics and the assessment period at each wave with all sample sizes.

**Table 1:** Information about data collection.

Wave	Response rate	Sample size (*N*)	% (*n*) women	Mean age (range)	Survey date	No. of days to complete the survey
1	100.0%	3,617	50.5% (1827)	47.6 (18–92)	April 6th–May 6th 2020	14
2	63.0%	2,282	48.9% (1115)	49.0 (18–86)	April 21st–May 13th 2020	7
3	65.5%	2,369	49.2% (1165)	48.8 (18–86)	May 4th–May 25th 2020	7
4	63.5%	2,296	48.5% (1113)	48.9 (18–86)	May 18th–June 10th 2020	7
5	59.6%	2,154	48.7% (1048)	49.3 (18–92)	June 1st–June 23rd 2020	7
6	58.5%	2,116	48.8% (1033)	49.4 (18–92)	June 15th–July 13th 2020	14
7	57.6%	2,072	49.1% (1017)	49.8 (18–92)	July 13th–August 8th 2020	14
8	51.7%	1,871	49.4% (924)	50.4 (18–92)	August 17th–September 13th2020	14
9	50.3%	1,821	48.4% (882)	51.8 (18–92)	September 21st–October 19th 2020	14
10	52.5%	1,883	48.4% (911)	50.3 (18–86)	November 25th–December 28th 2020	30

**Table 2:** Distribution of participants according to province of residence.

Wave	Province of residence									
	Alberta	British Columbia	Manitoba	New Brunswick	Newfoundland and Labrador	Nova Scotia	Ontario	Prince Edward Island	Quebec	Saskatchewan
1	12.1% (439)	14.7% (532)	3.3% (119)	2.0% (71)	1.4% (51)	3.4% (123)	39.1% (1415)	0.7% (25)	20.6% (747)	2.6% (95)
2	12.1% (276)	14.6% (334)	3.3% (75)	2.1% (47)	1.5% (34)	3.7% (85)	41.5% (947)	0.6% (14)	18.0% (411)	2.6% (59)
3	12.2% (289)	14.5% (344)	3.2% (76)	1.6% (39)	1.3% (31)	3.3% (79)	40.6% (962)	0.6% (15)	20.1% (475)	2.5% (59)
4	12.5% (287)	15.2% (348)	3.2% (74)	1.7% (40)	1.3% (30)	3.5% (80)	40.8% (937)	0.5% (11)	19.3% (443)	2.0% (46)
5	13.1% (283)	14.7% (316)	3.6% (77)	1.9% (41)	1.3% (27)	3.8% (81)	41.4% (891)	0.6% (14)	17.3% (372)	2.4% (52)
6	12.3% (260)	13.9% (294)	3.4% (72)	1.9% (40)	1.3% (27)	3.6% (76)	41.3% (874)	0.6% (12)	19.4% (411)	2.4% (50)
7	12.8% (266)	14.8% (306)	3.4% (71)	1.7% (35)	1.4% (28)	3.2% (67)	39.4% (816)	0.6% (13)	20.2% (419)	2.5% (51)
8	12.6% (235)	15.7% (293)	3.2% (59)	2.1% (39)	1.3% (24)	3.7% (69)	40.0% (749)	0.4% (8)	18.7% (350)	2.4% (45)
9	12.2% (222)	14.6% (265)	3.1% (57)	1.8% (33)	1.2% (21)	3.1% (57)	42.7% (778)	0.5% (9)	18.7% (341)	2.1% (38)
10	12.7% (239)	15.15 (284)	3.1% (58)	2.1% (39)	1.0% (18)	3.3% (62)	39.9% (751)	0.5% (9)	20.3% (383)	2.1% (40)

#### Participants

A total of 3,617 adults residing in Canada were recruited via the online survey firm *AskingCanadians* (*Qu’en pensez-vous*, in French). The sample is representative of the adult Canadian population by age, gender and province of residence at the first wave of the study (de la Sablonnière et al., 2020a). The proportion of participants born in Canada and abroad was also comparable to the Canadian population, with 79.5% (*n =* 2,858) of participants reporting being born in Canada. Regarding the proportion of Chinese people, 4.2% of participants indicated China as their country of birth (de la Sablonnière et al., 2020a), which was consistent with data from Statistique Canada (2013) indicating that Chinese people accounted for 4.0% of the Canadian population in 2011. More broadly, 6.7% (*n =* 243) of the sample considered themselves Chinese.

At wave 1, participants were aged between 18 and 92 (M = 47.6; SD = 17.0) and females represented 50.5% of the sample (*n =* 1,827; with 0.1% “other,” *n =* 2). The age of participants was distributed as follows: 11.0% aged 18–24 (*n =* 399), 16.6% aged 25–34 (*n =* 599), 16.9% aged 35–44 (*n =* 614), 15.9% aged 45–54 (*n =* 578) and 21.8% over 65 (*n =* 789). The level of education was particularly high within the sample since half of the sample (50.2%; *n =* 1815) had a university degree.

##### Exclusion criteria

First, as a precaution to ensure that the participation was taken seriously, we excluded participants who responded too quickly to a questionnaire but only for the wave in question (i.e., less than 4 min). This cut-off point was determined following the survey firm’s recommendation. No cut-off could have been set for extremely long completion time since participants had more than 1 day to complete the questionnaire and had the possibility to do it several times. We also introduced two attention check items from the second wave and excluded participants who failed to correctly answer at both for the wave in question. Second, we excluded participants who did not answer to at least three waves for the dependent variable (i.e., “prejudice,” starting from wave 3 to 10) as it is the minimum number of waves required for modeling a linear trajectory of change. Third, we excluded participants who identified themselves as “other” in terms of gender, who represented a negligible proportion of the sample (*n =* 2, 0.1%). To conclude, participants were excluded from the original sample based on these three criteria. The remaining sample consisted in 2,460 participants at wave 1.

##### Sample’s representativeness

The sample’s representativeness was established using the first wave of the survey (de la Sablonnière et al., 2020a). Although trajectories analysis uses FIML, missing data largely due to attrition may impact the results as well as the sample’s representativeness.

First, we conducted a binary logistic regression model to examine if some socio-demographic variables would predict individuals’ participation in the last 10th wave. Participants’ province of residence was not included in the logistic regression model because it made little sense to pick or select a particular province or region as the comparison group. The number of participants by province seemed stable from waves 1 to 10 (Table 2). According to the results (Table 3), only age is a significant, but weak predictor of participation in the 10^th^ wave, with older participants being more willing to have participated (*b* = 0.02, Wald *χ2(*1), = 32.13, *p* < 0.001). The conclusion of that analysis is that since attrition is not associated with participants’ socio-demographic characteristics, it should not have affected the sample’s representativeness over time.

**Table 3:** Coefficients for the binary logistic regression predicting participation at wave 10.

	Estimate	Standard Error	Value of *p*	Odd ratio	95% CI
Constant	−0.06	0.21	0.772		
Age	0.02	0.00	0.000	1.02	(1.01–1.02)
Gender	0.09	0.10	0.357	1.10	(0.90–1.34)
Education (university vs. non-university)	0.07	0.10	0.499	1.07	(0.88–1.31)
Political affiliation	0.01	0.03	0.582	1.02	(0.96–1.07)
Chinese ethnicity	0.22	0.19	0.238	1.24	(0.87–1.79)

*R*^2^ = 0.02 (Cox-Snell), 0.02 (Nagelkerke). Model *χ*^2^ (5) = 34.91, *p* = 0.000.

Second, in order to limit the disparity between our sample and Canada’s adult population, we carried out a weighting process (Mercer et al., 2018) to adjust for identifiable socio-demographic deviations of our sample, based on available data from Statistics Canada. The weighting process was conducted under the function “calibration” from the icarus package on R. The weighting process selected the best combination of calibration variables (among gender, province of residence, number of people in the household, number of minors in the household, Canadian born, Aboriginal origin, mother tongue and education) fitting model by retaining the one that minimized the average estimation error on a range of 13 external benchmark measurements based on data available from Statistics Canada. In short, the weighting procedure used tried to find the balance between reducing the bias due to the lack of representativeness of the sample and artificially increasing standard errors. The maximum range of the weights was fixed at 2.5 and the weighting procedure consequently allowed us to create weights ranging from 0.5231 to 3.0231 with a mean of 1. The weighting process reduced bias by 9.56% according to the selected benchmark variables. The analysis was performed with and without the weights. Since result patterns were the same, analyses including the weights are presented in order to improve the sample’s representativeness.

#### Procedure

The participants could choose which language (French or English) they preferred to complete the survey. Completion took between 15 and 20 min per questionnaire and was carried out online using an electronic device such as a cellphone, tablet, or computer. Participants who completed the first wave were invited to complete all subsequent ones. In other words, even if they missed one or more waves, they had the possibility to return to their participation at each new one. As a result, the number of waves to which participants responded may vary from participant to participant. Participants were rewarded with approximately 2.50 Canadian dollars per completed questionnaire obtained in the form of points redeemable at one of the partner companies of their choice.

### Measures

#### Objective 1: Trajectory groups of prejudice

##### Prejudice against Chinese people

From waves 3 to 10, participants were asked to report their personal feelings toward “Canadians” and “Chinese people” using a Likert scale, ranging from 1 (*extremely negative)* to 10 (*extremely positive*). Participants who preferred not to answer were given the option of choosing: “Prefer not to answer” in which case their answer was coded as missing.

To create a measure of prejudice against Chinese people, a “difference” score was used: the attitude toward Chinese people was subtracted from the attitude towards Canadians. Positive scores indicated higher prejudice against Chinese people, while a negative score indicated a prejudice in favor of Chinese people. A score of zero indicated that the participant judged Canadians and Chinese people alike, that neither group is better rated than the other. This method was used in previous studies to indirectly evaluate prejudice towards an outgroup, as it consists in a more appropriate measure of prejudice than simply assessing attitudes (Guimond and Palmer, 1993; Guimond and Dambrun, 2002; Levin et al., 2002; Guimond et al., 2003; de la Sablonnière et al., 2013). This method has also been used to evaluate prejudice in the context of COVID-19 (Zingora et al., 2021).

#### Objectives 2 and 3: Linking the trajectory group membership of prejudice with antecedents

##### Objective 2: Socio-demographic factors

At wave 1, participants were asked to report their age (open question), their gender (coded as 0 = *female*, 1 = *male*, or 2 = *other*), and their political affiliation using a Likert scale ranging from 1 (*strongly left)* to 10 (*strongly right*).

##### Objective 3: Psychological mechanisms

###### Economic threat

At wave 1, participants were asked to report their level of personal and collective relative deprivation using a Likert scale ranging from 1 (*extremely deteriorated)* to 10 (*extremely improved*). We adapted relative deprivation items widely used in the literature to the COVID-19 pandemic context (de la Sablonnière et al., 2010, 2013) such as: “Compared to before the COVID-19 pandemic, my economic situation has…” (personal relative deprivation) and “Compared to before the COVID-19 pandemic, Canadians’ economic situation has…” (collective relative deprivation). The items were then recoded so that a higher score indicated more economic threat.

###### Identity status

First, to measure the degree to which participants identified themselves as Canadians and global citizens, an adapted version of Cameron's (2004) identification scale was used. At wave 1, participants indicated their level of agreement using a Likert scale, ranging from 1 (*strongly disagree)* to 10 (*strongly agree*) to the following statements: “I think of myself as a Canadian” and “I think of myself as a global citizen.” Literature supports that identification can be adequately measured with a single item (Postmes et al., 2013; Reysen et al., 2013).

Second, to evaluate identity status, we examined the difference score between identification to Canadians and identification to global citizens to create a global–local identification score. The goal behind this measure is to assess the degree of inclusiveness based on the global–local continuum of identification strategy. The same formula as for the “difference” score measurement of prejudice was used to create the identity score: we subtracted identification with global citizens from identification with Canadians. Positive scores indicated that the participant’s identification strategy was more local (i.e., Canadian), while a negative score indicated the contrary, a global and more inclusive identification strategy (i.e., global citizenship identification). A zero score indicated that the respondent did not favor a particular identification strategy.

##### Control variable

###### Chinese ethnicity

Information about the ethnicity of participants was provided externally by the survey firm. Participants were asked to select one or more ethnicities from a large list. We controlled for Chinese ethnicity (coded as 0 = *non-Chinese*, or 1 = *Chinese*).

### Data analysis plan

#### Objective 1

The first objective of data analysis was to identify several trajectory groups of prejudice. Analysis were carried out using the PROC TRAJ procedure on SAS 9.4 software (Jones et al., 2001; Jones and Nagin, 2007), and the figures were generated with the function “ggplot” from the R package ggplot2. To evaluate objective 1, we used a semiparametric group-based modeling developed by Nagin (1999). The goal of this analysis is to identify who reports similar levels of change in prejudice over time from the overall sample groups of participants. Thus, the modeling of trajectory groups makes it possible to explore the intra-individual (e.g., over time) and inter-individual (e.g., between the identified sub-groups) change in a characteristic or a behavior. The trajectory groups were modeled with the censored-normal distribution (CNORM, Nagin, 1999) as the variables were continuous and followed a relatively normal distribution. To conduct a trajectory analysis, the first step requires determining the optimal number of trajectory groups with the proportion of participants in each trajectory group. The second step is to determine the optimal shape of each trajectory group (e.g., increase, decrease, stable, hump-shaped). We compared models ranging from one to six trajectory groups. The guideline to estimate trajectory groups advises that for a trajectory group to be sufficient at least 5% of the sample should be assigned to it. It is possible for larger samples such as ours to omit the 5% criteria, as long as each trajectory has at least 100 participants assigned to it (Frankfurt et al., 2016). The selection of the final model was based on the Bayesian Information Criterion (BIC) for which the value closest to zero indicates a better fit of the model with the data (Dunger et al., 1998; Nagin, 1999).

#### Objectives 2 and 3

The second and third objectives were to test the association between these trajectory group membership and antecedents: socio-demographic factors (i.e., age, gender, political affiliation) and psychological mechanisms (i.e., personal relative deprivation, collective relative deprivation, global–local identification). To test our hypotheses, we ran a multinomial logistic regression model. It allowed to estimate the probability of being assigned to each trajectory groups (compared to a reference trajectory group) based on individual-level factors. First, we entered all our antecedent variables in the model (i.e., age, gender, political affiliation, personal relative deprivation, collective relative deprivation, global–local identification). Second, we added the control variable (i.e., Chinese ethnicity) in the model.

#### Missing data

In trajectory groups analysis, missing data are handled via the Full Information Maximum Likelihood (FIML) method. Thus, that algorithm allows for the inclusion of participants who have missing data on the variables used to create the trajectory groups (Nagin, 1999; Jones et al., 2001). However, missing data for antecedent variables were not estimated and participants who had not answered all of the independent variables were automatically excluded from the analysis (Nagin, 1999). We did not have missing data on the antecedent variables since they were measured at wave 1 and participants were required to answer them to submit their surveys. However, there is missing data (*n =* 417, 16.95%) for the control variable (i.e., Chinese ethnicity) as it was assessed separately by the survey firm, so these participants were excluded from the regression analysis.

## Results

### Extreme scores and outliers

The assumption of normality of the variables is not required by mixed nonparametric models (Dupéré et al., 2007), but all variables were still normally distributed with skewness and kurtosis scores between +/−3 (Kline, 2016). Regarding univariate outliers, between 16 and 28 participants (0.9–1.4% of the sample for the wave in question) were identified as outliers on all eight variables of prejudice, and 17 (7%) on the collective relative deprivation variables. The Mahalanobis distance also identified 26 (1.06%) multivariate extreme scores.

The analyses were performed with and without extreme scores. To limit the potential impact that extreme scores may have had on results, univariate extreme scores were reduced to the limit of +/− 3.29 standard deviation and multivariate extreme scores were removed. Since their inclusion did not alter the results, participants with extreme scores (both univariate and multivariate) are included in the presented results.

### Descriptive and main analysis

Table 4 presents the means, standard deviations, and correlations between continuous variables.

#### Objective 1: Trajectory groups of prejudice

Four trajectory groups of prejudice were identified. Table 5 presents the BICs for the model selection based on the number of trajectory groups. A model with 6 groups did not have enough participants for one group, so we did not run models with more groups even if the BIC tended to be better as we added groups. Moreover, even if the BIC of the model with 5 groups was better than the BIC for the model with 4 groups, we chose with parsimony to keep 4 groups. Indeed, as we added a 5th group, the 4th group splitted into two groups following the same trajectory of change with little difference regarding the initial level of prejudice.

**Table 4:** Means, standard deviations, and correlation matrix.

Variables	Mean	Standard deviation	1	2	3	4	5	6	7	8	9	10	11	12	13
1.Age W1	50.04	16.72	—												
2.Political affiliation W1	5.20	1.94	0.08^**^	—											
3.Personal relative deprivation W1	6.25	1.83	0.07^**^	−0.01	—										
4.Collective relative deprivation W1	8.00	1.97	0.15^**^	−0.05*	0.35^**^	—									
5.Global–local identification W1	1.80	3.05	0.06^**^	0.11^**^	0.07^**^	0.08^**^	—								
6.Prejudice W3	1.17	2.18	−0.01	0.09^**^	−0.03	−0.03	0.14^**^	—							
7.Prejudice W4	1.12	2.20	0.07^**^	0.14^**^	0.01	0.01	0.20^**^	0.56^**^	—						
8.Prejudice W5	0.98	2.13	0.05^*^	0.14^**^	−0.02	−0.00	0.16^**^	0.54^**^	0.63^**^	—					
9.Prejudice W6	1.01	2.23	0.04	0.11^**^	−0.01	−0.02	0.17^**^	0.55^**^	0.63^**^	0.67^**^	—				
10.Prejudice W7	1.03	2.23	0.04	0.13^**^	−0.02	−0.02	0.18^**^	0.57^**^	0.66^**^	0.62^**^	0.67^**^	—			
11.Prejudice W8	0.95	2.17	0.02	0.16^**^	−0.03	0.01	0.18^**^	0.56^**^	0.65^**^	0.64^**^	0.67^**^	0.71^**^	—		
12.Prejudice W9	1.05	2.29	0.02	0.11^**^	0.00	0.01	0.17^**^	0.56^**^	0.60^**^	0.63^**^	0.66^**^	0.67^**^	0.71^**^	—	
13.Prejudice W10	0.89	2.12	0.03	0.13^**^	−0.01	−0.05	0.18^**^	0.50^**^	0.57^**^	0.59^**^	0.65^**^	0.63^**^	0.66^**^	0.67^**^	—

* *p* < 0.05;

** *p* < 0.01.

**Table 5:** BICs and probabilities for the selection of the 4-trajectory groups model.

No. of trajectory groups	BIC	Model probability	% of participants per trajectory group
1	−32623.84	0.00	100
2	−29724.19	0.00	86.0; 14.0
3	−28942.92	0.00	72.7; 21.8; 5.5
**4**	**−28438.67**	**0.00**	**4.9; 71.4; 18.5; 5.3**
5	−28275.21	0.00	4.4; 63.0; 20.5; 8.0; 4.0
6	−28228.14	1.00	2.8; 4.6; 62.6; 19.9; 8.0; 4.0

BIC = Bayesian information criterion. All trajectories were coded in cubic order. The bold values correspond to the selected model.

**Figure 1:**
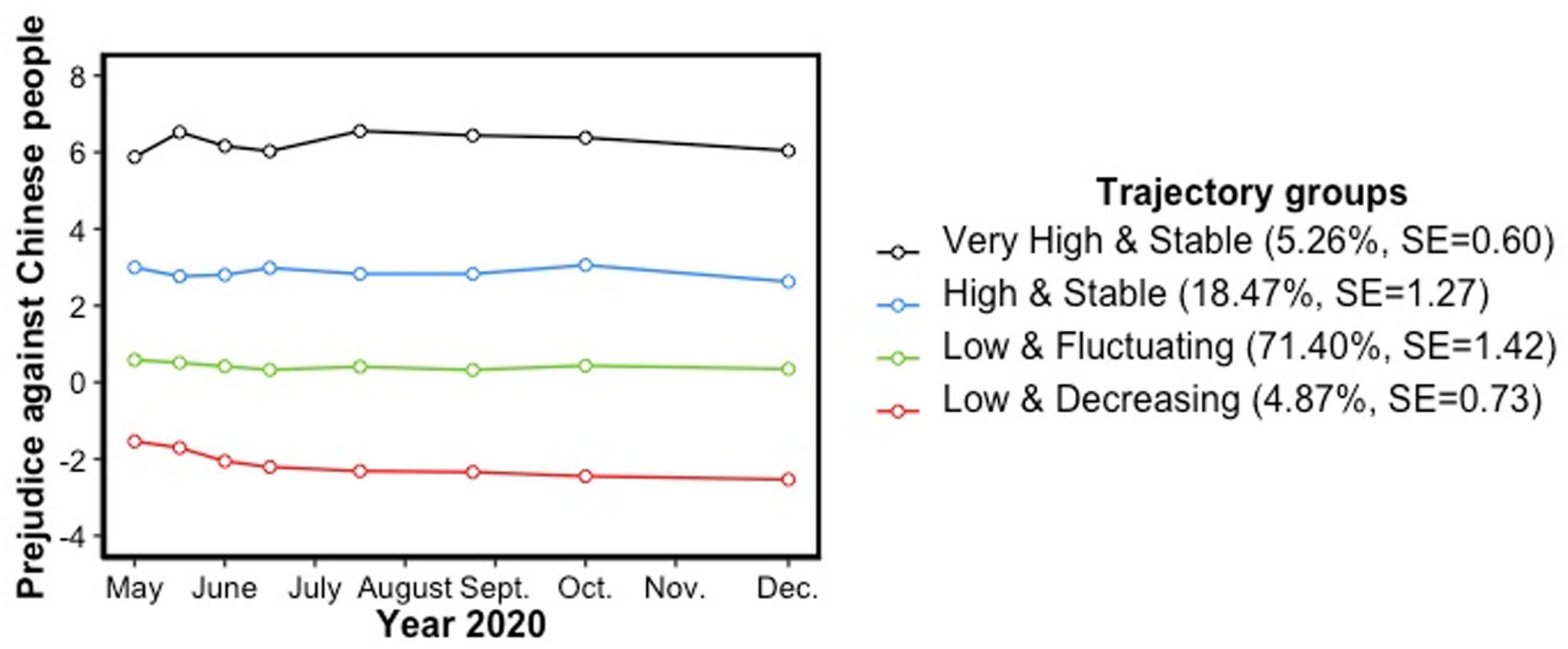
Trajectory groups of prejudice against Chinese people.

**Figure 2:**
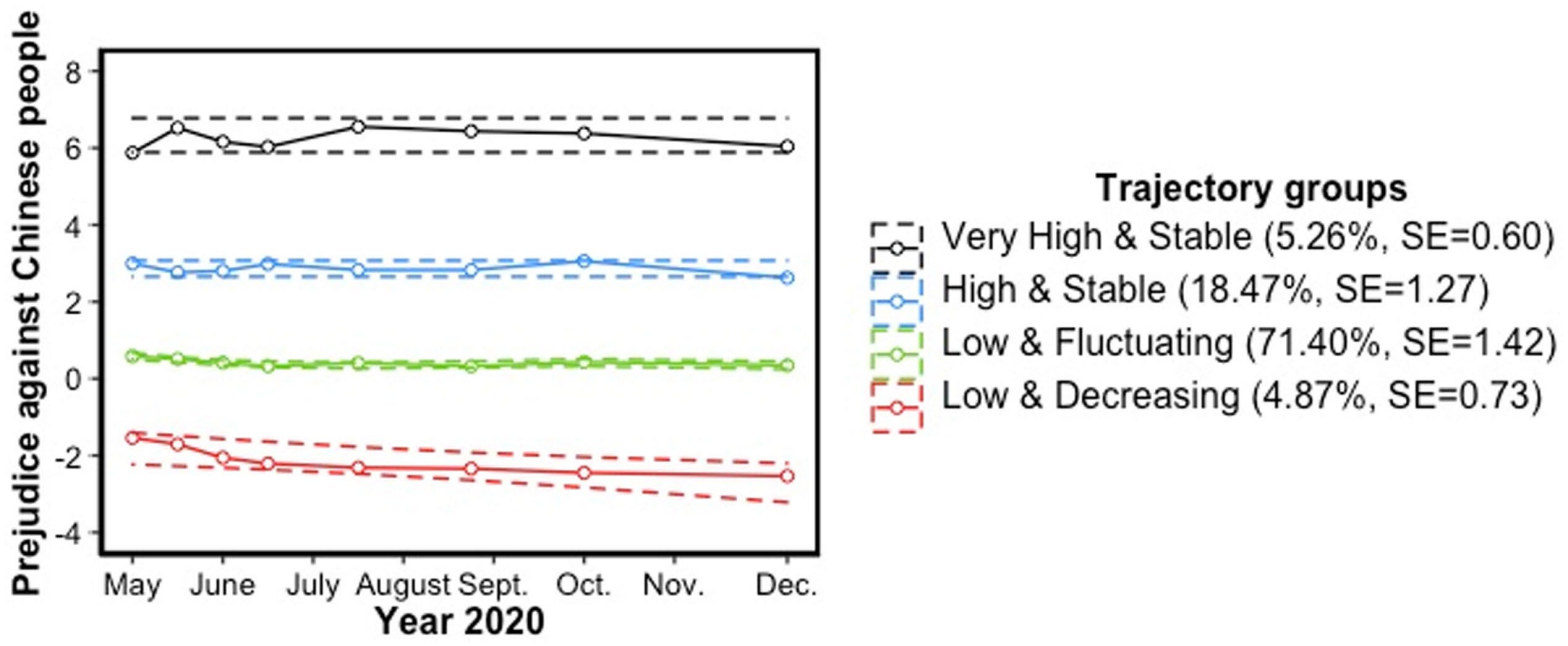
Trajectory groups of prejudice against Chinese people with confidence intervals.

Although trajectory groups looked relatively stable in their levels of prejudice against Chinese people from early April to late December 2020 (see Figures 1, 2), the model that best fitted the data revealed significant changes in prejudice over time. The parameters of the chosen model are presented in Table 6. Parameters of each trajectory group were determined using the BIC as well as their level of significance. This four-trajectory group model was made up of two trajectory groups that remained stable regarding their levels of prejudice over time (i.e., varying intercept order): a very high prejudiced trajectory group (5.26% of the sample; in black) and a high prejudiced trajectory group (18.47% of the sample; in blue). Otherwise, the majority of participants belong to a low prejudiced trajectory group (71.40%; in green) which shows a slight, but significant change in prejudice over time (i.e., cubic order), that is an inverted S starting by a decrease. Finally, a minority of participants (4.87%; in red) reported a better attitude towards Chinese people than Canadian people, that is to say they hold a low prejudice *in favor* of Chinese people over Canadian people, that becomes more positive over time (i.e., linear order).

**Table 6:** Coefficients estimates for the group-based trajectory model.

Trajectory group	Parameters	Estimate	Standard Error	value of *p*
1. Low and decreasing	Intercept	−1.82	0.21	0.000
	Linear	−0.03	0.01	0.003
2. Low and fluctuating	Intercept	0.59	0.04	0.000
	Linear	−0.06	0.01	0.000
	Quadratic	0.00	0.00	0.000
	Cubic	−0.00	0.00	0.001
3. High and stable	Intercept	2.28	0.11	0.000
4. Very high and stable	Intercept	6.36	0.18	0.000
	Sigma	1.51	0.03	0.000

Labels of these trajectories were described according to two characteristics. First, the starting level of prejudice; second, the stability or malleability of its evolution over time. Thus, the four trajectory groups were, respectively, labelled and classified: 1) “Low and Increasing” (in red), 2) Low and Fluctuating” (in green), 3) “High and Stable” (in blue), and 4) “Very High and Stable” (in black). It can be noted that the low levels of prejudice of the first trajectory group (i.e., in red) that will be interpreted in this paper as a low prejudice in favor of Chinese people could also be considered as having a low prejudice against Canadian people which would thus increase over time (due to our operationalization of prejudice in terms of a difference score in attitudes).

#### Objectives 2 and 3: Linking the trajectory group membership of prejudice with antecedents

Antecedents of trajectory group membership are divided into socio-demographic factors (objective 2) and psychological mechanisms (objective 3). Antecedents were all entered in the multinomial logistic regression model. Then, the control variable was added.

Results are reported in Tables 7, 8. The probabilities of membership for each trajectory group were estimated compared to a reference group. The second trajectory group (“Low and Fluctuating” in green), served as a reference group for two reasons: first, because of all trajectory groups, its level of prejudice is closest to 0 and can consequently provide a neutral baseline. Second, because it is the majority group.

**Table 7:** Antecedents of trajectory group membership of prejudice.

Variable	Trajectory group (ref. group = 2)	Estimate	Standard Error	Value of *p*	Odd Ratio	95% CI
Constant	1	−2.77	0.90	0.002	0.06	(0.01–0.37)
	3	−2.57	0.43	0.000	0.08	(0.03–0.18)
	4	−3.20	0.74	0.000	0.04	(0.01–0.17)
Age	1	−0.02	0.01	0.001	0.98	(0.96–0.99)
	3	0.00	0.00	0.357	1.00	(1.00–1.01)
	4	−0.00	0.01	0.971	1.00	(0.99–1.01)
Gender	1	0.52	0.28	0.060	1.68	(0.98–2.89)
	3	0.17	0.14	0.232	1.19	(0.90–1.57)
	4	0.11	0.24	0.644	1.12	(0.70–1.77)
Political affiliation	1	−0.13	0.09	0.138	0.88	(0.75–1.04)
	3	0.15	0.03	0.000	1.16	(1.09–1.24)
	4	0.16	0.08	0.034	1.18	(1.01–1.37)
Personal relative deprivation	1	0.22	0.08	0.008	1.24	(1.06–1.46)
	3	0.01	0.04	0.794	1.01	(0.93–1.09)
	4	−0.01	0.08	0.901	0.99	(0.85–1.15)
Collective relative deprivation	1	0.04	0.08	0.661	1.04	(0.88–1.22)
	3	−0.01	0.04	0.792	0.99	(0.92–1.07)
	4	−0.09	0.07	0.196	0.92	(0.80–1.05)
Identity status	1	−0.18	0.06	0.005	0.84	(0.74–0.95)
	3	0.08	0.02	0.000	1.08	(1.04–1.13)
	4	0.17	0.05	0.000	1.19	(1.08–1.29)

CI, Confidence interval; Trajectory groups 1, Low and Decreasing; 2, Low and Fluctuating (reference group); 3, High and Stable; 4, Very High and Stable.

**Table 8:** Antecedents of trajectory group membership of prejudice with the control variable.

Variable	Trajectory group (ref. group = 2)	Estimate	Standard Error	Value of *p*	Odd Ratio	95% CI
Constant	1	−3.61	1.20	0.003	0.03	(0.00–0.28)
	3	−2.52	0.45	0.000	0.08	(0.03–0.19)
	4	−3.52	0.87	0.000	0.03	(0.01–0.16)
Age	1	−0.02	0.01	0.113	0.98	(0.97–1.00)
	3	0.00	0.00	0.647	1.00	(0.99–1.01)
	4	0.00	0.01	0.463	1.00	0.99–1.02)
Gender	1	0.40	0.31	0.205	1.49	(0.81–2.74)
	3	0.20	0.16	0.214	1.22	(0.89–1.66)
	4	0.00	0.26	0.987	1.00	(0.61–1.66)
Political affiliation	1	−0.12	0.11	0.299	0.89	(0.71–1.11)
	3	0.16	0.04	0.000	1.17	(1.09–1.26)
	4	0.20	0.08	0.017	1.22	(1.04–1.44)
Personal relative deprivation	1	0.19	0.09	0.040	1.21	(1.01–1.46)
	3	0.02	0.04	0.605	1.02	(0.94–1.11)
	4	−0.05	0.09	0.541	0.95	(0.80–1.12)
Collective relative deprivation	1	0.06	0.10	0.529	1.07	(0.88–1.30)
	3	−0.01	0.04	0.740	0.99	(0.91–1.07)
	4	−0.05	0.08	0.553	0.95	(0.82–1.11)
Identity status	1	−0.17	0.08	0.028	0.84	(0.72–0.98)
	3	0.08	0.02	0.000	1.09	(1.04–1.14)
	4	0.16	0.05	0.001	1.17	(1.07–1.28)
Chinese ethnicity	1	1.70	0.38	0.000	5.47	**(**2.58–11.59**)**
	3	−0.90	0.46	0.051	0.41	(0.16–1.00)
	4	−1.67	0.71	0.019	0.19	(0.05–0.76)

CI, Confidence interval; Trajectory groups 1, Low and Decreasing; 2, Low and Fluctuating (reference group); 3, High and Stable, 4, Very High and Stable.

##### Objective 2: Socio-demographic factors

We hypothesized that age, gender and political affiliation would be associated with trajectory group membership (H2). While there was no sub-hypothesis for age and gender, age showed significant association, but only with one trajectory group: more specifically, younger participants were more likely to belong to the “Low and Decreasing” (in red) compared to the “Low and Fluctuating” (in green) trajectory group than older participants (a 2% increase in probability per younger year, odds ratio [OR] =0.98; 95%CI = 0.96–0.99; *p* = 0.001). Gender was not significant.

Regarding political affiliation, results supported that conservatives would be more likely to belong to high trajectory groups of prejudice than liberals (H2a). More specifically: participants were 16% more likely to belong to the “High and Stable” (in blue) compared to the “Low and Fluctuating” (in green) trajectory group as they reported 1 extra point towards a more conservative affiliation (OR = 1.16; 95%CI = 1.09–1.24; *p* = 0.000). The percentage was up to 18% when comparing the “Very High and Stable” (in black) and the “Low and Fluctuating” (in green) trajectory groups (OR = 1.18; 95%CI = 1.01–1.37; *p* = 0.034). Difference between the “Low and Decreasing” (in red) and the “Low and Fluctuating” (in green) trajectory groups was not significant.

##### Objective 3: Psychological mechanisms

###### Economic threat

We hypothesized that participants who felt more economically threatened would be more prejudiced (H3). More specifically, it was expected that participants who reported more personal (H3a) and collective (H3b) relative deprivation would be more likely to belong to high trajectory groups of prejudice. Results did not support our hypotheses H3a and H3b, as the more participants reported personal relative deprivation, the more they were likely to belong to the “Low and Decreasing” (in red) compared to the “Low and Fluctuating” (in green) trajectory group (a 24% increase in probability for each extra point on the personal relative deprivation scale; OR = 1.24; 95%CI = 1.06–1.46; *p* = 0.008). Other trajectory group comparisons were not significant. Regarding collective relative deprivation, results were not significant either. In other words, the more participants felt economically threatened the more they were likely to belong to the trajectory group of prejudice which favor Chinese people over Canadian people. Moreover, this result only stems from personal threat since there were no trajectory group difference regarding collective threat.

###### Identity status

We also hypothesized that participants who identified more locally instead of globally would be more likely to belong to high trajectory groups of prejudice (H4). Results supported our hypothesis: the more participants favored a local over a global strategy of identification, the more they were likely to belong to the “High and Stable” (in blue) compared to the “Low and Fluctating” (in green) trajectory group (an 8% increase for each extra point towards the local strategy; OR = 1.08; 95%CI = 1.04–1.13; *p* = 0.000). This percentage was up to 19% when comparing “Very High and Stable” (in black) and “Low and Fluctuating” (in green) trajectory groups (OR = 1.19; 95%CI = 1.08–1.29; *p* = 0.000). Finally, participants were 19% less likely to belong to the “Low and Decreasing” (in red) compared to the “Low and Fluctuating” (in green) trajectory group as they reported one extra point towards a more local (over a global) strategy of identification (OR = 0.84; 95%CI = 0.74–0.95; *p* = 0.005).

##### Control variable

Chinese ethnicity was added in the model as a control variable to examine if participants who considered themselves as Chinese were more likely to belong to certain trajectory groups than non-Chinese participants, especially the “Low and Decreasing” (in red) trajectory group. When controlling for Chinese ethnicity, age was no longer associated with trajectory membership. Otherwise, results showed that when comparing to the “Low and Fluctuating” (in green) trajectory group, Chinese participants were 547% more likely to belong to the “Low and Decreasing” (in red; OR = 5.47; 95%CI = 2.58–11.59; *p* = 0.000) and 52.6% (OR = 0.19; 95%CI = 0.05–0.76; *p* = 0.019) less likely to belong to the “Very High and Stable” (in black) trajectory groups than non-Chinese participants. Differences between “High and Stable” (in blue) and “Low and Fluctuating” (in green) trajectory groups were not significant.

### Additional analysis: Removing Chinese participants from the sample

The goal of these additional analyses was to examine if results are replicated when participants of Chinese ethnicity are removed from the sample (*N* = 2,287). The model that best fitted the data was the same four-trajectory groups model regarding the number of groups and the shape of each trajectory group (Supplementary Tables 1, 2; see Supplementary material). Supplementary Figures 1, 2 (see Supplementary material) showed that trajectories of prejudice looked similar over time. The distribution of participants in each trajectory group was also quite similar (with a loss of approximately 1% participants in the “Low and Decreasing” trajectory group; in red). Regarding the multinomial logistic regression model for association with antecedents, the patterns of results were relatively similar (Supplementary Table 3; see Supplementary material). There were two differences: first, the association between personal relative deprivation and trajectory group membership (occurring between the “Low and Decreasing” and the “Low and Fluctuating” trajectory groups) was not significant anymore. Second: regarding identity status, there were no longer any significant differences between the “Low and Decreasing” (in red) and the “Low and Fluctuating” trajectory groups (in green).

## Discussion

The aim of the present study was to deepen our understanding of change in prejudice against Chinese people within the adult Canadian population during the COVID-19 pandemic. Previous studies using a long-term perspective focused on the dynamic of prejudice, with a general approach where the population was considered as a homogeneous entity. Other correlational or experimental studies investigated the antecedents of prejudice, regardless of their dynamics over time. Overall, the literature suggested a general increase in prejudice during the first months of the pandemic, pointing out some antecedents linked to prejudice. Yet, no study explored if this observed increase in prejudice is truly embraced by everyone in a given population, or inversely, if there is a possibility for individuals to be divided into several subgroups which follow a different trajectory of change in prejudice over time. Our focus on the inter-individual heterogeneity regarding change in prejudice addressed the limitation of the general approach used by long-term perspective studies. At the same time, it addresses the limitations of cross-sectional and experimental studies regarding their lack of interest toward the dynamics of prejudice while maintaining the study of antecedents of change in prejudice as secondary objectives.

### Prejudice trajectory groups

The first objective was to identify different trajectory groups of prejudice within a large, representative sample of the adult Canadian population by age, gender and province of residence, from May 2020 to December 2020. According to the first hypothesis, the Canadian population has been shown to be heterogeneous regarding their initial levels of prejudice and, to a lesser extent, their variations over time. Four trajectory groups of prejudice relatively stable over time were identified. Specifically, we observed during the investigated period that about a quarter of the Canadian population had a stable, high or very high prejudice against Chinese people during the pandemic. However, most Canadians (approximately 70%) had low levels of prejudice which fluctuated according to the results, but looked, in fact, very stable over time. Besides, changes in prejudice for this trajectory group were characterized by very small effect sizes which could be attributed to the large proportion of participants assigned to this group. Finally, a minority of Canadians (less than 5%) reported low prejudice in favor of Chinese people which became more positive throughout 2020. Not surprisingly, individuals who considered themselves as Chinese were more likely to belong to this “Low and Decreasing” trajectory group, just as they were less likely to belong to the “High [or] Very High and Stable” trajectory groups of prejudice against Chinese people, compared to the “Low and Fluctuating” trajectory group against Chinese people. Nevertheless, future national-level studies on prejudice should consider including members of minority groups as they are part of the population in everyday life, whether they emigrated or were born citizens of different ethnic origins. Just as the exclusion of Chinese participants in the present study did not erase the “Low and Decreasing” trajectory group which favor Chinese people over Canadian people, there is a possibility for individuals, particularly of minority groups, to internalize prejudice against their own ingroups (Kite and Whitley, 2016).

Regarding the literature on prejudice against Chinese people during the pandemic, the present study did not replicate any tendency towards an increase in prejudice over the first months of the pandemic since no trajectory group followed this pattern of change. On the contrary, we observed that low prejudiced individuals displayed a slightly lower level of prejudice over time, while high prejudiced individuals remained stable over time. Overall, the trend of the trajectory groups looked nevertheless very constant over time which did not support the strong resurgence of the international “anti-Chinese sentiment” in the Canadian population (Rad, 2020). It also suggested that even if prejudice is malleable, it seemed highly resistant to change even in such a social, dramatic context. This resistance to change, especially in the case of high prejudice, supports attitudinal research which shows that more extreme attitudes are more likely to be crystallized than more “centered” attitudes (Eagly and Chaiken, 1995). Besides, change in prejudice could have occurred before our prejudice’s data collection gathered from May 2020, since previous studies suggested an increase in prejudice following the first months of the pandemic (Vachuska, 2020). Finally, there is also a possibility that change in prejudice against Chinese people differed by countries. Thus, even if Chinese Canadians were also facing more racism since the pandemic (Rad, 2020), the stability (and the decrease for one group) in prejudice observed in the present study could be explained by a cultural “shifting blame.” Thereby, Hirsch et al. (2022) found that if Canadians first considered the Chinese as being responsible for the pandemic, they rather gradually blamed people who did not respect the sanitary measures over the course of the first months. Future studies could focus on prejudice against several groups to investigate how it varied simultaneously.

### Antecedents of prejudice trajectory groups

The second and third objectives were to associate trajectory group membership of prejudice with antecedents: socio-demographic factors (i.e., age, gender, political affiliation) and two psychological mechanisms (i.e., economic threat, identity status).

After the identification of different trajectory groups of prejudice, the second most important result of the present study related to the link between identity status and trajectory group membership of prejudice. With our original operationalization of identity status based on a global–local continuum, we showed that participants were more likely to have higher and stable prejudice against Chinese people as they favor a local over a global strategy of identification. Thus, the present study corroborates the literature regarding the assumption that local identification is positively associated with the highest level of prejudice while global citizenship identification is negatively associated with prejudice (Castano et al., 2002; McFarland, 2017; Sparkman and Eidelman, 2018; Bourhis, 2020; Jetten et al., 2020). Nonetheless, the literature distinguishes two forms of local identification while considering a national level (de Zavala et al., 2009; Kervyn et al., 2015; also see Marchlewska et al., 2022). The secure national identity is positive and characterized by national pride and openness toward outgroup members. The narcissistic national identity, related to an exaggerated belief about the national ingroup’s greatness and a rejection of outgroup members, is negative since it is generally associated with detrimental intergroup outcomes such as lower intergroup forgiveness (Hamer et al., 2018), greater intergroup aggressiveness and prejudice (de Zavala et al., 2009; de Zavala and Lantos, 2020). It might also influence how we collectively respond to major social issues, as the study of Bertin et al. (2021) shows for example that national collective narcissism predicts a lower acceptance of climate science through a greater endorsement of climate change conspiracy beliefs. Future studies should consider the dual conception of identification while investigating prejudice to better understand how and why these processes are related.

The second psychological mechanism proposed in the present study is economic threat and has shown an unexpected association with trajectory group membership of prejudice. Results did not support the hypothesis that participants who felt more economically threatened will be more likely to belong to high trajectory groups of prejudice. We found that economic threat was only related to low trajectory groups of prejudice and predicted the membership in the “Low and Decreasing” compared to the “Low and Fluctuating” trajectory group. Moreover, this association concerned personal (but not collective) economic threat as assessed by relative deprivation. Nevertheless, the association between personal relative deprivation and trajectory group membership of prejudice only concerned the “Low and Fluctuating” trajectory group, which is over-represented by Chinese people. Besides, results became non-significant when Chinese participants were removed from the analysis. In this perspective, we could argue that this surprising result related specifically to Chinese people included in our sample. A possible interpretation could thus be that Chinese people would more likely be (increasingly) prejudiced against Canadian people as they perceive a deterioration of their economic situation due to the pandemic. Such an interpretation would better fit the scientific literature which supports that the more individuals felt economically threatened the more they report prejudice.

Apart from this result, the absence of a link between economic threat and prejudice contradicts previous studies. For example, Dhanani and Franz (2021) experimentally demonstrated that American people facing severe economic threat in the pandemic context were more prejudiced against Asian people than American people feeling mildly economically threatened. A possible explanation of such contradictory results could be attributed to the study context. Specifically, the eventual means put in place by each country to address the economic impact of the pandemic could compensate for the economic losses eventually experienced by its population. In the United States, although there were some governmental financial supports for the population at the beginning of the pandemic, the management of the crisis by Trump’s administration would have led to an “immense economic pain and an increase in social inequality” according to The New England Journal of Medicine (Kolata, 2021, para. 9). Comparatively, the Canadian Federal Government came up with a multitude of financial support programs for individuals, communities, businesses, and sectors such as tourism and transport, plus financial assistance from provincial governments (Government of Canada, 2022) making it possible to ensure that no one in Canada was left behind. This rapid and supportive response to its population may have helped to create an atmosphere of social cohesion and economic security that in turns may explain that the perception of personal or collective economic deterioration was not associated with the evolution of prejudice against Chinese people in our study. Future studies are needed to understand why there are contradictory results, exploring, for example, which factors moderate the link between economic threat and prejudice in a cross-national setting.

Another explanation could be proposed in line with the relative deprivation theory. Although literature widely supports that relative deprivation is associated with prejudice (Smith et al., 2012), the study of Guimond and Dambrun (2002) did not systematically find a link between relative deprivation and prejudice. Rather, Guimond and Dambrun (2002) found that relative gratification could be even more related to prejudice than relative deprivation. Several subsequent studies support the existence of a V-curve hypothesis (Dambrun et al., 2006; Anier et al., 2016; Eller et al., 2020). Future studies could use two distinct measures, by exploring, for example, whether participants report more prejudice as they perceive either an improvement (relative gratification) or a deterioration (relative deprivation) in their economic situation compared to before the pandemic.

Regarding socio-demographic antecedents, results were also mixed. First, the sub-hypothesis which presumed that conservatives compared to liberals would be more likely to belong to the highest trajectory group of prejudice was supported, replicating previous studies (Tsai et al., 2020; Dhanani and Franz, 2020a, b, 2021). Nevertheless, recent literature recognizes that both liberals and conservatives can be prejudiced but against different groups, as prejudice would be a result of the perception of ideological divergences rather than a matter of political affiliation *per se* (Kite and Whitley, 2016). Then, future studies could investigate which groups are prejudiced depending on each political affiliation, how these prejudices evolved during the pandemic, and examine if individuals react the same way based on their political affiliation (e.g., discrimination).

Second, while no sub-hypothesis was made for age and gender, results suggested that younger people were more likely to belong to the “Low and Decreasing” compared to the “Low and Fluctuating” trajectory group (in green), but this difference became non-significant when controlling for Chinese ethnicity. Generally, associations between age and prejudice have never been well supported in the literature (Allport, 1979). Even studies on prejudice against Asian people during the pandemic found contradictory results: some demonstrated that the older people were the more prejudiced they were against Asian people (Dhanani and Franz, 2020a, b, 2021), while some demonstrated that younger people were the more prejudiced they were against Asian people (Tsai et al., 2020). Otherwise, age and prejudice may be linked by a much more complex than a linear association. For example, Henry and Sears (2009) found some evidence for an inverted V-curve of prejudice in adulthood with a peak around middle age. Thereby, if developmental theories of prejudice in childhood are well documented (see Kite and Whitley, 2016), those in adulthood still need further investigations.

Finally, we did not find that women or men were more likely to belong to certain trajectory groups of prejudice than other trajectory groups. Then, the study suggested that gender might have nothing to do with the levels of prejudice, at least within the Canadian population, particularly in the case of prejudice against Chinese people.

### Implications, limitations, and futures directions

The present study has two main implications. As a first theoretical implication, the present study demonstrates the importance of considering inter-individual differences when it comes to understanding prejudice and its change over time. The use of group-based modeling, to our knowledge, has never been used in the study of prejudice against Chinese people during the context of the COVID-19 pandemic. This methodological choice constitutes a major and original contribution of the present study. It allowed us to deepen our understanding of previous studies by addressing the question: Did a given population report the same trajectory of prejudice against Chinese people over the course of the pandemic?

The consideration of inter-individual differences also applied while we investigated the antecedents of prejudice. A second theoretical and practical implication of the present study results from the exploration of antecedents associated with the different trajectory groups of prejudice. By demonstrating that some socio-demographic categories are more likely to belong to high and stable trajectory groups of prejudice (i.e., individuals of conservative political affiliation), the present study informs governments and practitioners about who could benefit from awareness and interventional efforts to prevent prejudice against Chinese people during the pandemic. As suggested by Litam (2020), behind the victims of discrimination, there are prejudiced perpetuators who need to be educated. Most importantly, the present study provides suggestions in terms of application. It demonstrates that relying on and promoting a more global and inclusive identity instead of a local one is a promising way to harmonize interpersonal and even international relationships. This is particularly relevant in the context of dramatic social change, which is characterized by societal and individual changes so profound that they might lead to an identity threat (e.g., changes in the group’s values, attitudes, behaviors) and consequently impact intergroup relationships (de la Sablonnière, 2017; Cárdenas and de la Sablonnière, 2020).

Taken together, these two main implications are even more relevant as we consider that the present study is the first Canadian one to provide empirical data on prejudice against Chinese people during the pandemic. In addition, one of the main strengths of this study is to benefit from a large and representative sample of the adult Canadian population by age, gender and province of residence, that allowed the generalization of results to the entire studied population. On the one hand, the present study specifically addressed Canadian circumstances (e.g., governmental, public health), which may benefit from such empirical data regarding its population. On the other hand, the present study contributes to the international literature on prejudice against Chinese people during the pandemic, which is relevant as countries differ in terms of prejudice (Guimond et al., 2013; Guimond, 2019; de la Sablonnière et al., 2020b).

Despite its contributions, the present study has nevertheless certain limitations. First, we chose to focus on prejudice against Chinese people because of the anti-Chinese rhetoric related to the COVID-19 pandemic. Yet, several authors claimed that Chinese and Asian people were not the only minorities at risk to be targets of prejudice and discrimination since the onset of the pandemic (Lund, 2020). The study of Vachuska (2020) demonstrated, for example, that the COVID-19 pandemic has also been associated with an increasing “anti-Hispanic sentiment.” Therefore, future studies should investigate which social groups have been the most impacted during the pandemic in terms of intergroup relationships and which ones need social support.

A second limitation of the present study relates to our late assessment of prejudice, which started in May 2020. In the present study, the different trajectory groups of prejudice identified looked relatively stable over the course of the pandemic. Such results do not replicate previous literature that suggests an increase in prejudice against Chinese people during the first months of the pandemic (Nguyen et al., 2020; Schild et al., 2020; Vachuska, 2020). Yet, anti-Chinese and anti-Asian racism have also been reported in Canada since the pandemic (Rad, 2020). Future Canadian studies are needed to compare prejudice against Chinese people before and after the pandemic. For example, correlational studies could use a direct, retrospective measure of prejudice. Researchers could also conduct qualitative studies among Canadian participants to deeply explore the dynamic of prejudice against Chinese people before and during the ongoing pandemic.

The assessment of political affiliation also constitutes a limitation of the present study. The liberal–conservative continuum has been used as a single item by several studies focusing on prejudice (Pettigrew and Meertens, 1995; Tsai et al., 2020) and even on political ideology (Van Leeuwen and Park, 2009; Calvillo et al., 2020), suggesting that it consists in an adequate explicit measure of political affiliation as it leads for example to the same results as an implicit measure of political affiliation (Van Leeuwen and Park, 2009). Nevertheless, the use of the liberal–conservative continuum has been criticized since it relates to concepts (e.g., “liberal” or “left”) which evokes different representations and appears to be very abstract for most individuals (Bauer et al., 2017). In the same vein, Wojcik et al. (2021) found that the liberal–conservative continuum (referred as “left” and ‘right” in their studies) endorses different meanings for Eastern and Western European participants. In addition, Wojcik et al. (2021), assessed two dimensions of liberalism: cultural (or social) and economic, and found them to be differently associated with the liberal–conservative continuum. Since previous studies also showed evidence that considering these different dimensions of political affiliation has important implications on outcomes of interest (Carney et al., 2008; Cichocka and Jost, 2014), further studies could benefit from using it. The use of multiple items rather than a single one would help to improve the fidelity and validity of the measurement tool, but more importantly it would allow to explore the complexity of political affiliation.

Finally, although the present study benefited from a large, representative sample of the Canadian population regarding age, gender and province of residence, it still should be mentioned as a fourth limitation that some groups such as high-educated individuals are over-represented in the sample (in Canada 28.5% of the population has a university degree whereas in our sample that proportion reached 54.3% in the first wave of the study; see de la Sablonnière et al., 2020a). Although we addressed the impacts of this limitation by relying on a weighting process to adjust for such socio-demographic deviations, readers should keep this information in mind when considering the conclusions of the present study.

## Conclusion

Since prejudice is a natural and universal intergroup bias, it is widespread in human relationships. There is even more prejudice against certain groups in times of crisis such as during wars, starvation or epidemics. As prejudice increases in intensity, it may turn into negative actions from antilocution to extreme mass extermination (Allport, 1979). Thus, prejudice constitutes a major social issue which needs to be addressed by asking the right questions. In the COVID-19 pandemic context, previous studies investigated whether there was an increase in prejudice against Chinese people or they examined which factors led to this prejudice. The present study kept these two concerns in mind but came up with the original perspective of inter-individual heterogeneity. We hope that this report regarding the Canadian population and prejudice against Chinese people during the COVID-19 pandemic and their antecedents will offer useful information and solutions to political and public health authorities, as well as offering researchers new theoretical and methodological perspectives.

## Data availability statement

The raw data supporting the conclusions of this article will be made available by the authors, without undue reservation.

## Ethics statement

The studies involving human participants were reviewed and approved by The University of Montreal’s research ethic committee in education and psychology. The participants provided their written informed consent to participate in this study.

## Author contributions

VF contributed to the research idea, statistical analysis, and preparation of the manuscript was under the theoretical and methodological supervision of ÉL, RS, and MP-D. ÉL, AD, MP-D, J-ML, DS, and RS was responsible for the project programming and implementation. All authors contributed to the article and approved the submitted version.

## Funding

This research project was funded by the Canadian Institutes of Health Research (CIHR) and by Mitacs.

## Conflict of interest

The authors declare that the research was conducted in the absence of any commercial or financial relationships that could be construed as a potential conflict of interest.

## Publisher’s note

All claims expressed in this article are solely those of the authors and do not necessarily represent those of their affiliated organizations, or those of the publisher, the editors and the reviewers. Any product that may be evaluated in this article, or claim that may be made by its manufacturer, is not guaranteed or endorsed by the publisher.
