# Supplementary material for: COVID-19, economic threat and identity status: Stability and change in prejudice against Chinese people within the Canadian population
Source: Front Psychol. 2022 Oct 28;13:901352. doi: 10.3389/fpsyg.2022.901352 (PMC9650986; doi:10.3389/fpsyg.2022.901352)
Supplement: Supplementary file 1 [file Data_Sheet_1.pdf]

Supplementary Material

1 Supplementary Figures and Tables

1.1 Supplementary Figures

Supplementary Figure 1. Trajectory groups of prejudice against Chinese people (Chinese participants excluded

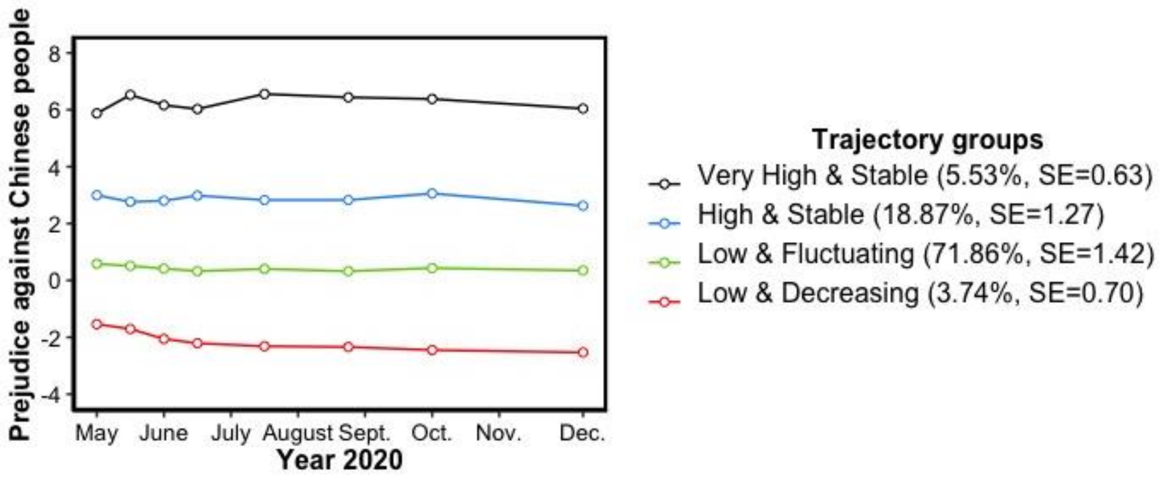

Supplementary Figure 2. Trajectory groups of prejudice against Chinese people with confidence intervals (Chinese participants excluded).

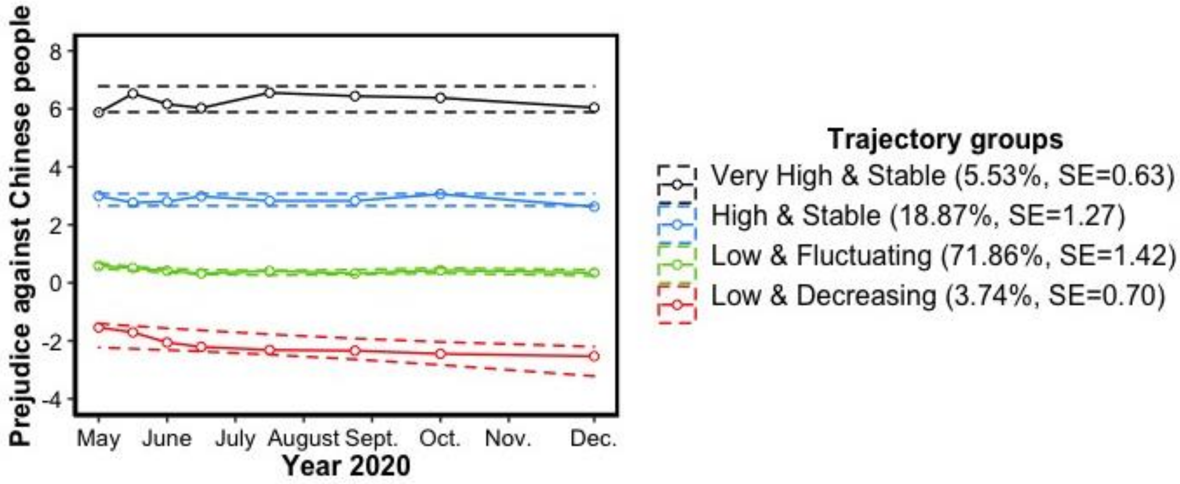

1.2 Supplementary Tables

Supplementary Table 1. BICs and probabilities for the selection of the 4-trajectory groups model (Chinese participants excluded).

No. of trajectory groups	BIC	Model probability	% of participants per trajectory group
1	-30253.39	0.00	100
2	-27483.57	0.00	85.6 ; 14.4
3	-26737.56	0.00	73.2 ; 21.2 ; 5.7
**4**	**-26355.07**	**0.00**	**3.7 ; 71.8 ; 18.9 ; 5.5**
5	-26214.88	0.00	3.2 ; 62.9 ; 20.8 ; 8.6 ; 4.6
6	-26168.48	1.00	0.2 ; 3.8 ; 64.8 ; 20.0 ; 8.2 ; 3.0

Supplementary Table 2. Coefficients Estimates and Error terms for the selected model (Chinese participants excluded).

Trajectory group	Parameters	Estimate	Standard Error	*p-value*
1. Low and Decreasing	Intercept	-1.79	0.27	0.000
	Linear	-0.04	0.01	0.005
2. Low and Fluctuating	Intercept	0.61	0.04	0.000
	Linear	-0.06	0.01	0.000
	Quadratic	0.00	0.00	0.000
	Cubic	-0.00	0.00	0.001
3. High and Stable	Intercept	2.91	0.10	0.000
4. Very High and Stable	Intercept	6.38	0.17	0.000
	Sigma	1.51	0.03	0.000

Supplementary Table 3. Antecedents of trajectory group membership of prejudice **(**Chinese participants excluded).

Variable	Trajectory group (ref. group = 2)	Estimate	Standard Error	*p-value*	Odd Ratio	95% CI
Constant	1	-2.42	1.03	.019	0.09	(0.01 - 0.67)
	3	-2.50	0.44	.000	0.08	0.03 - 0.20)
	4	-3.04	0.76	.000	0.05	(0.01 - 0.21)
Age	1	-0.02	0.01	.035	0.98	(0.96 - 1.00)
	3	0.00	0.00	.656	1.00	(0.99 - 1.01)
	4	-0.00	0.01	.717	1.00	(0.99 - 1.01)
Gender	1	0.42	0.31	.183	1.52	(0.82 - 2.80)
	3	0.19	0.15	.202	1.21	(0.90 - 1.61)
	4	0.13	0.24	.592	1.14	(0.71 - 1.83)
Political affiliation	1	-0.14	0.10	.169	0.87	(0.71 - 1.06)
	3	0.16	0.03	.000	1.18	(1.10 - 1.26)
	4	0.17	0.08	.025	1.19	(1.02 - 1.38)
Personal relative deprivation	1	0.16	0.11	.130	1.18	(0.95 - 1.45)
	3	0.01	0.04	.722	1.01	(0.94 - 1.10)
	4	-0.00	0.08	.975	1.00	(0.86 - 1.16)
Collective relative deprivation	1	0.00	0.10	.977	1.00	(0.83 - 1.22)
	3	-0.02	0.04	.643	0.98	(0.91 - 1.06)
	4	-0.10	0.07	.120	0.90	(0.79 - 1.03)
Identity Status	1	-0.16	0.08	.061	0.86	(0.73 - 1.01)
	3	0.07	0.02	.001	1.07	(1.03 - 1.12)
	4	0.17	0.04	.000	1.18	(1.08 - 1.29)

Note. CI = Confidence interval.
Trajectory groups 1 = Low and Decreasing, 2 = Low and Fluctuating (reference group), 3 = High and Stable, 4 = Very High and Stable.
